# Municipal Restriction of Disease1Read before the Quarter-Centennial Celebration of the Michigan State Board of Health and the Conference of State and Provincial Boards of Health of North America, Detroit, Mich., August 10, 1898.

**Published:** 1898-10

**Authors:** Ernest Wende

**Affiliations:** Buffalo, N. Y.; Professor of dermatology, Medical Department, University of Buffalo, and Health Commissioner of the City of Buffalo; 471 Delaware Avenue


### BUFFALO MEDICAL JOURNAL.

VOL. XXXVIII. OCTOBER, 1898. NO. 3

### Original Communications.

### MUNICIPAL RESTRICTION OF DISEASE.[1]

BY ERNEST WENDE, M. D., Buffalo, N. Y.

Professor of dermatology, Medical Department, University of Buffalo, and Health Commissioner of the City of Buffalo.

IT IS more than eighteen hundred years ago since there was a set of men who "thought they ought to be heard for their much speaking." As they have propagated exceedingly since that time, and as I observe that they flourish just now to a surprising extent everywhere, I will do my best to avoid adding to that prolific race.

You will remember in Paul Clifford that that very sagacious person August Tomlinson said "Life is short and why should speeches be long?" An aphorism so sensible under all circumstances, and particularly in the circumstance in which I am placed, I shall practically adopt on the present occasion. Therefore, no time need be occupied in rehearsing before this audience the necessity of municipal action against contagious diseases and other adjunct factors that imperil the well-being of the community. Such diseases and factors are of vital concern to every public body, to every individual and to every household as a household and in proportion as it is appreciated so in proportion an enlightened public demands for itself as high a degree of protection as may be attained, without contesting its humane significance and civilising influence.

If contagious diseases are preventable, why do they continue to exist? Why not prevented? Why not diminished with the progress of sanitary science and civilisation? In reply largely because of the apathy, ignorance and unconsciousness on the part of the public of what may befall them, and because of the indifference, mismanagement and conflicting interests on the part of the local health authorities in

1. Read before the Quarter-Centennial Celebration of the Michigan State Board of Health and the Conference of State and Provincial Boards of Health of North America, Detroit, Mich., August 10, 1898.

combatting infection. Until the laity better appreciate the benefits resulting from a willing and intelligent obedience to Nature's laws of how to live and avoid disease, until they recognise the fact that without their assistance, the efforts of the sanitarian to control contagion and to blot out sickness, sorrow and suffering, are of little avail, but little radical improvement can be brought about.

Then, what is necessary to consummate such an end? (1) The education of the masses in all that pertains to public hygiene, to enlist their coöperation against carelessness and disregard in the application of laws or principles for the preservation of health in whatever way they may be employed. (2) Municipal equipment and organisation to carry out modern sanitation, to be of such intelligence and activity as to make it a blessing to the people and creditable to a progressive city. (3) Strict ordinances, rules and regulations, the execution of which should be arbitrary, prompt and impartial. (4) Laws obtained from the legislature of the state giving plenary powers to the health authorities in matters of municipal sanitation.

What should the ordinances embody? (1) A complete classification of the recognised infectious diseases, with a specific definition of the same, for legal guidance and vindication of public rights. (2) Mandatory notification on the part of the physician or householder, through the public telephone system, immediately upon discovery of any contagious malady specified by law, for the action of the municipal health officer. (3) The placarding of infectious houses as signals of danger, for public warning. (4) Isolation at home and prohibition from school attendance, from exposure in public places, or from any other aid in spreading contagion, also the rigid enforcement of quarantine when indicated, in order to secure the full benefit of isolation. (5) The proper removal and reception of infected persons, when necessary, to a suitable hospital. (6) Compulsory vaccination and revaccination for self-protection and for the benefit of others. The failure to provide and enforce this safeguard against small-pox, to allow its work of death and disfigurement unimpeded is maliciously wicked and criminally neglectful. (7) The sanitary disposition of the dead from infectious and pestilential diseases. (8) Thorough cleansing and disinfection of the infected house and its contents under municipal direction, and the destruction of bedding, clothing and other articles exposed to infection, also the prohibition of throwing any infectious material or rubbish into any receptacle for the deposit of refuse without previous disinfection. (9) Supervision over the erection of all tenement and lodging houses, to insure a standard amount of light, ventilation, plumbing, cubic air space and other hygienic features and by systematic inspection to so maintain them. (10) School-house inspection by medical inspectors, with power to regulate and advise concerning school-room sanitation, plumbing, sanitary propositions, personal hygiene, exercise and physical culture, contagious diseases and the like. (11) The creation of small parks in connection with public schools, thus affording suitable school environment, ample play-ground, breathing space in the crowded district, and a corresponding beneficial influence upon the developmental period of youth and childhood. (12) Thorough inspection of food products, of dairies and cows and prohibition of the sale of infected milk. (13) The establishment of free public baths, to be located in the manufacturing centers and in sections where the poor predominate. Frequent baths are invigorating tonics, which restrict disease, promote health, morality and self-respect. (14) An enactment to furnish labor for the unemployed poor "who, through neglect, are allowed to become sick, a public charge and hence a double expense," as industry gives force and vigor to life, conduces to health and contentment, which means municipal economy. (15) The establishing of a bacteriological laboratory in furnishing available assistance in the diagnosis of diphtheria, tuberculosis and typhoid fever, and in assisting us to form a rational idea respecting disinfection, isolation and the prevention and cure of diseases generally. (16) A systematic dissemination of detailed directions and hygienic rules for the special needs of unsanitary features and conditions of any kind or nature, and their application judiciously insisted upon. (17) Rigid enforcement of the penalties for violation of all sanitary laws, and especially those relating to infectious diseases and their control. (18) Regulations and rules defining the relation of the medical practitioner to the medical officer of health, regarding their respective duties to infectious diseases. (19) Qualifications of the highest grade in medical officers of health who should possess technical education, tact, firmness and fearlessness, and who should be selected on account of fitness. The municipality should not be exposed to an unnecessary risk by politics. It should override it. (20) Arrangement for keeping the health department open on every day in the year, and at all hours—day and night—to receive reports of infectious diseases so that no time may be lost in taking such immediate action as the emergency demands.

The scope of this paper precludes the consideration of each proposition in detail. However, as a type for the purpose of elucidation and demonstrating the phases in the development of measures adopted for the restriction of diseases in Buffalo, the three maladies—diphtheria, as representing the contagious and infectious; tuberculosis, combining contagion with heredity; infantile diarrhea, as illustrating the diatetic and hygienic, will best serve the object.

*Diphtheria.*—The nature of diphtheria, its possibilities and long recognition has attracted the efforts of the sanitarian and physician, so that today the most glorious triumphs in the reduction of mortality has been achieved by the employment of preventive measures. Thus based upon our present sanitary knowledge, we combat it upon the subjoined principles. (*a*) That it is a contagious, virulent malady, capable of enormous injury and dependent upon a specific organism, resident in the throat or other locality attacked. (*b*) That under certain procedures, if possible to carry forth to a successful end, it could practically be stamped out.

The group of acute infectious diseases, of which diphtheria is a representative, demands and should receive the most aggressive action. While ever present, its limitations has step by step progressed, been circumscribed, and this has been and can be accomplished in communities upon the following lines:

First and most important, the prompt recognition of the true nature of the malady. No affection has been the subject of more difference of opinion in diagnosis by the rank and file of practitioners. In many cases, pending the correct diagnosis of the disease, great havoc has been wrought, while, on the contrary, the enormous assumption of its existence has entailed anxiety, economic damage, and worse, either a distrust of correct diagnosis or apathy toward its possibilities.

With the possibilities of accurate diagnosis by bacteriological tests, it is now incumbent upon all properly protected municipalities to have in operation and quickly available, the resources of an effective laboratory that, without delay, the profession can confirm or dismiss cases in doubt. The value of this method is now no longer questioned by the most extreme practitioner, and to a progressively enlightened public, interested in sanitary matters, acquiescence in it is accomplished, if not demanded by the public at large.

The system in vogue in Buffalo represents what is believed to be as practical and stringent as circumstances will permit. It is obligatory upon physicians to report to the department of health (at all times) by telephone, which is free of expense to them, each case of diphtheria immediately upon discovery as it comes under their notice. At least twenty-four hours is saved by the telephone and notification of the public by placarding the house is accomplished just so much sooner than reporting to the department by mail; thus possibility of further spread of the disease is materially reduced. If doubt as to the nature of the malady exists, the physician has at his command, in all sections of the city, the resources of the laboratory and a report within twenty-four hours. This procedure is made more available by the coöperation of the police department. In each police station of the city, culture tubes and swabs, compactly put up in a small box, with printed directions for inoculating, so simple that a novice is capable of following them, can be had by any physician. The culture outfit contains a blank to be filled out by the physician, furnishing the laboratory with the name of the patient, address, duration of disease, location of membrane and such other information as the department records require for a full knowledge of the case. Through this same channel the culture outfits are returned to the health department, passed upon and within twenty-four hours or less, the medical man is furnished with a diagnosis of his case and the house placarded if necessary, without further cognisance on his part. Through this system of coöperation between the police and health departments the service is kept entirely under municipal control and rapidity and freedom from error made definite. The placard is maintained until a bacteriological examination demonstrates the absence of the diphtheria bacilli.

The placarding of the house for the public information is immediately followed by a visit from a sanitary inspector who institutes such other sanitary measures as seem indicated. A detailed report upon the premises is presented by the inspector, including all such features as defective plumbing, unhygienic surroundings, possible source of the infection, together with such recommendations as, in his judgment, the conditions demand; the department immediately ordering rectification of all sanitary defects. Further aggressive action is instituted following the inspector's report, notably notification of school principals of all cases occurring in their districts and thus interdicting school attendance by pupils from quarantined houses, and notification of public libraries that the circulation of infected books may be prevented.

The source of milk is also noted and the possibility of this medium as the infectious channel considered. On account of the importance of the milk supply as a possible source of infection, a special ledger is preserved in the department office, detailing each milk dealer's route, families supplied, source from which milk is obtained and such other data as may be necessary. This ledger is posted daily and thus the healthfulness of the families supplied by each individual dealer is constantly watched. As soon as it appears that on the route of any milk-dealer infectious disease exists, an immediate investigation is made into every detail of this business, source of supply and conditions existing there; when, if justification appears to exist or even a doubt, his route is suspended until it can be made absolute that his is not the source of danger. Furthermore, the dealers are not allowed to leave at, or remove from, an infected household any milk receptacle, neither can a milk-dealer fill a milk bottle on his wagon. The feature of the milk-dealer's routes and contagious disease supervision cannot be too strongly commended. It has demonstrated its importance several times in Buffalo in ascertaining and determining that to be the source of infection and made possible the arrest of several rapidly spreading epidemics, notably of scarlet and typhoid fever.

In addition to the above, each placarded household is furnished with a circular printed in the language of the occupants, setting forth the nature of the disease in question, laws pertaining to quarantine and instructions for the care of the sick, disinfection of excreta and detailed instructions to prevent further spread of the disease.

The feature of municipal placarding is the one mostly complained of on the part of the infected household, and one which in many cases determines dishonesty on the part of the attending physician and is a frequent source of much trouble to those concerned. The external evidence of the internal infection is thoroughly appreciated by those for whom benefit is intended, and almost as thoroughly objected to by those within. More particularly is this feature in evidence when placarding interferes with business, as when it occurs in connection with a store, or where other children in a household have to suspend their school life and practically be quarantined within. These are cogent reasons to the average family and when the moral sense of the physician is elastic and the interests of the household itself made supreme, concealment is attempted, often successfully, and the objects of sanitation defeated. So much injury or loss to small stores has occurred, so much resentment at interruption of routine household work has been occasioned, that in certain sections of the city it was, at one time, equivalent to loss of practice for a medical man to diagnosticate and announce diphtheria. Thus, unfortunate conflict between interest and sense of duty has been in evidence in house placarding for all contagious diseases and it is to be regretted that there appears to be no feasible way at present to exact official action in interdicting it. In many cases of this character, the health department has been able to detect and punish in court such violations of law, through complaint or information furnished by an interested neighborhood. This is not, however, to be construed as evidence of a high sense of duty, but rather as an illustration of the dependence upon the question of "whose ox is being gored."

The features of bacteriological laboratory assistance, house placarding, expert sanitary house inspections and investigations, surveillance of milk routes and supplies are of supreme importance and efficiency. Each has its difficulties, but, notwithstanding, they are insisted upon and enforced. To the efficiency of enforcement the limitations of the contagious diseases have been narrowed and epidemic visitations made a matter of history. If to the factors enumerated could be added a system of compulsory official and municipal disinfection, it would almost appear that extermination of acute contagious diseases was in sight. It is remarkable that municipal equipment for municipal disinfection, the most cogent of measures, should yet be subsidiary.

To disinfect as now practised in most communities is a matter of department recognition carried out under the directions of the physician, with such implied efficiency as only the personal factors admit. Disinfection, to be efficient, should be obligatory, arbitrary, made by those specially trained and by up-to-date equipments and applied with intimate thoroughness of knowledge regarding the various agents and their particular use and indications in individual cases. The Department of Health should supervise the disinfection, see that contagion is annihilated and a public record maintained of such inspection and disinfection. From such record, data should be available for the real estate interests, for the public and for house hunters.

What is more pitiable, and, yes, more preventable, than the possibility of a family of little ones moving into a dwelling, perchance to be the victims of a pestilential infection? Medical men are but too familiar with instances of this character and its results.

We have to consider next the most formidable foe of the human race—tuberculosis—and one presenting propositions as difficult as important. A notable feature pertaining to it, and beyond question, is its prevalence amongst lower animals. No disease equals its fatality nor attacks so many of them. Domestic animals only vary in susceptibility and those escaping under natural conditions promptly respond and succumb when inoculated. Its communicability and identity in man and the lower animals, its enormous mortality and the methods of dissemination, with equally accurate knowledge as to the methods which could restrict its prevalence, directs criticism to what is largely left undone by the state, whose attitude in general is one of negligence or indifference.

The cause of this apathy need not detain us; it is explainable by lack of knowledge, experience, conflicting interest, sentiment and the like. In some sections, partial efforts have been and are made, yet the essential of success—uniform and systematic procedure—is lacking and until this is obtained, without considering the question of the ways and means, compensation and the like, the state which should be, if anything, prodigal in its expenditure for life and health, should maintain the following: (1) Quarantine against the introduction of tubercular cattle. This should be as rigid as that against any pestilential disease in man. Security against foreign invasion circumscribes the conditions and concentrates aggressive action at home. (2) Milk herds and dairy interests should be under strict surveillance and preferably under the license system. No dairyman should be permitted to engage in supplying milk without first obtaining a state license to do so, which should only be issued after inspection. Such inspection should include physical examination of the herds and the use of tuberculin test, together with consideration of all sanitary features bearing upon the animals and protection of milk. Buildings should be adapted to the business, the employees free from disease; care of animals, food, water, details of cleanliness, transportation features, and in fact all procedures, should be scrutinised and maintained up to a high standard of sanitary excellence. (3) No beast should be used for food without satisfactory evidence of its being free from disease. In cities, the public abbatoir system should prevail. The advantages of this are well known. It directs and concentrates the slaughter industry, permits inspection of all animals before killing and of the carcass and viscera after, and practically can be made to preclude the possibility of the consumption of unfit meat.

Of the transmission of tuberculosis by meat or the degree to which the flesh of tubercular cattle is fit for use there is some honest difference of opinion. There can be no question, however, that when the infection is more or less general and the animal in poor condition that such meat should be interdicted.

Literature pertaining to sanitary matters and particularly in the premises under consideration, should be freely extended until those to whom directed can know that sanitary excellence is to their highest advantage and interest.

With exclusion from without and extermination within, with a sanitary standard of sanitary excellence, mandatory, and all that it implies, one prolific source of infection is within reach of state action.

In the absence of such a protection as outlined and of jurisdiction, the Department of Health of Buffalo has adopted, under my direction, a scrutiny, incomplete as it may be, which briefly is as follows :

A record is kept of the conditions that prevail at each dairy supplying the city with milk. This information is obtained solely by inquiry, but has been found generally reliable. It includes data of the size, health, tuberculin examination of the herds, character of the water, food, health and employees, methods of milking, cooling, cleansing, transportation, in fact all factors bearing upon the purity and protection of the milk. Additionally, circulars are caused to be sent, bearing upon the possibilities, and the like, of the business. When conditions at the dairy appear prejudicial to the public health from any cause, investigation is made and correction requested.

In the absence of authority beyond the municipal limits, when action for any cause appears necessary for unsanitary reasons, for failure to comply with the department's request, the product of such dairy is interdicted at the city line and the dealer to whom consigned notified, until such time as the offense is removed.

With this mode of procedure and its results, any city can exercise a large influence upon dairies supplying it, exclude milk from known tubercular herds and obtain sanitary protection beyond its own field of authority.

With brief reference to this recognised source of tubercular infection and the outline of action toward its destruction, the more difficult and the more important question of how to deal with the tubercular consumptive presents itself. The consumptive being dangerous, largely from his habits, to those who incur danger from ignorance or necessity in these directions, sanitary knowledge is more cogently indicated. The personal element, the chronic nature of the malady; sentiment, social, religious, economic commercial bearing, all exercise and influence and create a public opinion that prohibits a course of procedure toward it that is directed toward other contagions. It, therefore, is left us to enforce what we can and thus far and in no mean degree bring about such good results as is possible.

Reporting by physicians and registration by the health department of all consumptives, their residences and other facts is reasonable, proper and practical, and permits such action by the authorities, with the coöperation of the physician, as can be exerted. By this registration it is possible to cause the dissemination of information of vital interest for the protection of the family and the public. It is needless to specify; briefly, it should relate to the disposition of the sputa, disinfection and should reach those for whom intended through the physicians in attendance, who can adapt, modify and secure the greatest efficiency with the least offense to the individual and household. It permits proper recording, and every health department should, from such registration, maintain a register of all homes, dwellings and the like subject to such infection, record its duration and date of satisfactory disinfection. Such register should be available to the public and those contemplating a change of house, for their guidance and protection, and it should, by ordinance, be incumbent upon owner or lessee to institute thorough disinfection under heavy penalty.

The disgusting nuisance of indiscriminate expectoration in street cars, streets and public places should be prohibited by law and made more efficient by creating public sentiment against it. The difficulty of enforcing such an ordinance is apparent, therefore the necessity of a strong public coöperation.

Hotels, boarding, tenement and lodging-houses should have special information and modes of procedure supplied them, that will enable them to adopt the principles of prevention or minimising the possibility of infection to the peculiarities of their business.

The street car conductors should enforce the company's rules regarding spitting upon the car floors with vigor, and every car floor should be properly cleaned with suitable disinfecting fluids. Cuspidors in public places should contain a disinfecting solution. Employees in crowded places, as factories, workshops and similar places should be subject to physical surveillance and offending cases weeded out. Sanitary rules regarding cubic air space, rules regulating expectoration, all cogent in the extreme, should be enforced.

If it is indicated to prevent the introduction into the state of tubercular cattle, so much more is it important to exclude all persons suffering from tuberculosis pulmonalis. To this end, then, entrance should be interdicted and uniformity of action in all sections of the country aimed at.

The emigration of tubercular patients in the breaking-down stage should be interdicted or discouraged, not only on account of the futility of it in the majority of cases, but from the fact of their disseminating contagion. No action toward the extinction of tuberculosis in communities would be complete if it did not take cognisance of the factors bearing upon those predisposed.

With this object, the broad field of general attitude to the health and physical development of the young, particularly in the crowded district, should receive careful attention. This implies supervision of crowded dwellings and placing them upon a healthful standard, school hygiene and calisthenics, public parks and the well-known details too numerous to mention. Attention is likewise necessitated to penal institutions and the like, where, under the influence of crowding, sedentary life and mental depression, this malady is prone to develop.

Public hospitals should have isolation wards and not be permitted to segregate the tubercular in wards with other medical cases. Preferably, hospitals for the special care of this class of patients should be provided and equipped with the particular features necessitated by the peculiarities of the malady and the moral of the patient.

The establishment of state hospitals for consumptives is now receiving the attention its importance demands. In Germany these institutions have been in successful operation for years. Massachusetts is about opening the first in this country and will be followed by one in New York State at an early day. There is every reason why the state should extend its humanitarian action towards this scourge as towards lunacy.

Tuberculosis among the poor is disastrous in its results, bringing untold misery to families and pauperism to the state. One source of infection frequently overlooked is the possibility of disseminating sickness through the dealers of second-hand clothing and household effects. Every large city has many of these establishments and without doubt they are frequently the receptacle of cast-off clothing, bedding and the like, from infected households. While, perhaps, difficult in every case to control this condition of affairs as at present, there can be no doubt it would be a wise procedure to supervise this traffic by ordinance and license.

*Diarrhea.*—The diarrheal diseases of infancy prevail with large mortality among all infants, but particularly among the poor, and are justly classified as preventable diseases, as they are due to toxic bacteria. The appreciation of this fact determines the action of health officials toward the prevention of food contamination the source of infection, inasmuch as these maladies are in the main limited to children who have not the advantage of breast milk.

In searching for removable causes of milk infection, none seems more cogent than the so-called long-tube nursing bottle. It is likely well known to all, and consists of the usual form of bottle with the addition of a rubber tube one foot and over in length ending in a nipple, the adaptation being made to permit self-feeding on the part of the child, thus obviating the necessity of an attendant, a device for a lazy mother or nurse who is unfitted for her sacred trust.

Investigation has shown an enormous proportion of diarrheal diseases in children artificially fed and using this form of contrivance not found with the use of others. This explanation is found in the fact that it is impossible to keep this tubing clean or safe. The mother, usually uneducated in the premises, cannot, by scalding or washing, maintain the inner surface of the tubing in condition so as not to contaminate the milk drawn through it.

I have conducted a series of experiments and investigations in the matter, the details of which I need not detain you with, the result of which, briefly, may be summarised as follows: The inner surfaces of these tubes are found to be irregular, present porosities and the seams are frequently imperfect. These indentations become the rendezvous of colonies of microörganisms, which thrive on the milk which stagnates in them and which cannot be dislodged by any ordinary process of cleansing. As a result of this condition, milk, receptive of infection by nature, aided by artificial warmth, becomes an easy media for the development of toxins.

At my suggestion the City of Buffalo has, by ordinance, prohibited the sale and use of these death-dealing bottles, though not without incurring the animus of certain commercial interests which have combined and are arrayed to combat the ordinance. It is needless to say that the profession has, without question, given its hearty support to the stand taken by the department and that the issue will be carried to a finish. If ordinances created to correct so patent a cause of infantile mortality can be set aside for commercial reasons, health laws in the future will have small chances for their existence.

Municipal protection of the milk industry constitutes a large and most important agent in diminishing the cause of infantile mortality. To secure this end, city milk houses should answer to the following principal requirements: (1) Proper light, air and ventilation. (2) Constructed of nonabsorbent material. (3) Without direct communication to sleeping apartments, water closets or any unsanitary room. (4) Use for milk storage solely. (5) Storage and cooling boxes metallic lined, elevated and placed to permit thorough cleansing. (6)

Sanitary plumbing, outside box ventilation, indirect drainage. (7) Sanitary rules posted conspicuously pertaining to cans and protection of milk. (8) Prohibition of the use of disinfectants—any necessity for their use implying unsanitary condition. (9) Prohibiting the leaving of bottles or receiving the same from any house placarded for contagious diseases and prohibiting milkmen from refilling or filling bottles on wagons or while en route.

The elimination of pernicious influences upon diarrheal diseases of infancy by maintaining a high degree of excellence in all that bears upon it would be incomplete without the coöperation of mothers in carrying out in the home-life the principles of infant hygiene. To secure this to the household containing a newborn child, the Buffalo Department of Health furnishes a circular of information, covering the essential points which mothers should observe in the care and management of the infant. That this may reach and have its influence upon all classes, the matter is printed in several languages, is couched in simple words and the directions given plainly and practical.

Such circulars of instruction should, and, in Buffalo, include the following: (1) Directions for bathing and cleanliness, for properly dressing and changing the infant. (2) Directions for sleep, regulating the hours and urgently warning against the use of sleeping drops, cordials and the like. (3) Directions for securing abundance of fresh air, suitable hours for airing the child and the importance of avoiding the devitalising effects of the heat within doors and without. (4) Directions for simple household hygiene, as regards ventilation, dampness, odors, and the like. (5) Directions, in detail, for feeding, weening, various artificial foods. The preservation and sterilisation of milk, methods of cleaning and keeping clean feeding bottles, utensils and the like. The selection of proper feeding bottles and condemnation of the long-tube design. (6) The symptoms readily recognised by mothers necessitating medical attendance and the evidence of normal digestion.

It is gratifying to know that these circulars are appreciated among the poorer classes, are lived up to as much as possible and have a salutary influence in the premises.

The principles involved in the subject under consideration have of necessity been but referred to and are intended as suggestive and to bring out the views and experiences of those among you who have given these matters much thought. The field is a most inviting one and it is through such exchange of views as is possible here that present action and procedures can be fortified and new ones made operative.

Sanitary science has been and is so progressive that any action that will proportionately annihilate the various contagions will redound to the happiness and welfare of the community and the honor of our art.

471 Delaware Avenue.

